# Tobacco use among blue-collar workers in Nigeria: a survey of construction artisans in Ekiti State

**DOI:** 10.11604/pamj.2025.50.70.38555

**Published:** 2025-03-11

**Authors:** Charles Oluwatemitope Olomofe, Hadii Mamudu, Kabir Adekunle Durowade, Oluwafunmike Ruth Olomofe

**Affiliations:** 1 Department of Health Science and Public Health, St Bonaventure University, St Bonaventure, New York, United States; 2 Department of Community Medicine, Federal Teaching Hospital Ido-Ekiti, Ekiti-State, Nigeria; 3 Department of Health Services Management and Policy, East Tennessee State University, Johnson City, United States; 4 Department of Community Medicine, College of Medicine and Health Sciences, Afe Babalola University, Ado-Ekiti, Ekiti State, Nigeria; 5 Department of Social Work, East Tennessee State University, Johnson City, Tennessee, United States

**Keywords:** Blue-collar workers, construction artisans, Tobacco

## Abstract

**Introduction:**

the prevalence of tobacco use among blue-collar workers such as construction artisans is disproportionately higher than in the general population, yet very limited studies have been conducted about such workers in Nigeria, the most populous country in sub-Saharan Africa. This study aims to assess the prevalence and the associated risk factors of tobacco use among construction artisans in Ekiti State, Nigeria.

**Methods:**

between November 2018 and January 2019, an adapted semi-structured questionnaire from the Global Adult Tobacco Survey was administered to collect data from 232 construction artisans. A multi-stage stratified sampling technique was employed to select participants, including carpenters, and bricklayers, journeymen, and their apprentices who were working in Ekiti State. Descriptive statistics, Chi-square, and logistic regression were conducted to delineate factors associated with tobacco use in this population.

**Results:**

all respondents were males. The prevalence of people who have ever smoked was 19.3%. Multivariate analysis showed that artisans who were within the age range of 31-40 years were four times more likely to use tobacco (aOR: 3.41, 95% CI: 1.48-7.88; p= 0.003) compared with people younger than 20 years. Additionally, being in school (aOR: 2.01, 95% CI: 1.03-3.93; p= 0.039) and being divorced/separated (aOR: 4.24, 95% CI: 1.31-13.76; p= 0.01) were associated with tobacco use, compared with the respective populations.

**Conclusion:**

majority of the respondents said there was no smoking restriction at the worksite and this may be an impetus to continue smoking. Therefore, smoke-free worksite regulations would be needed to curtail the smoking tendencies of these workers.

## Introduction

The construction industry plays a major and significant role in the employment creation and economic growth of many countries [1]. As high as 80% of people who work in the informal sector in Nigeria [1] are blue-collar workers, and construction artisans constitute a major segment of this population [1]. The construction industry in Nigeria also contributes significantly to the Gross Domestic Product (GDP) of the country [1,2]. Construction artisans are those who perform skilled work relating to the erection or assembly of large structures such as buildings, roads, and bridges with their hands [3]. These blue-collar workers are generally equipped with vocational education, which may be acquired formally or informally through observation, apprenticeship, and short learning cycle [4]. World Health Organization predicted that tobacco use among adults in Nigeria will increase by 5% by 2025 [5]. The prevalence of tobacco use among blue-collar workers has been reported to be disproportionately higher than in the general population in many countries [6]. In Morocco, the prevalence of tobacco use among blue-collar workers was 28.5% compared with 21% among white-collar counterparts [7]. Similarly, in India, smoking prevalence was 23.5% and 19.2% among blue-collar (production) and white-collar (non-production) workers, respectively [8].

Furthermore, in the United States (U.S.), compared with all other occupational groups, construction workers had the highest number of ever-smokers (48% versus 39% for all other occupations combined) [9]. Similarly, a survey of determinants of tobacco use among African countries revealed that unskilled (blue-collar) workers had higher risk of smoking than professionals (white-collar) workers and unemployed people [10]. The findings from these countries indicate the critical need to investigate the prevalence and understand the underlying protective factors for high rate of smoking in blue-collar workers in a country like Nigeria where majority of its informal sector workers are blue-collar workers. Construction artisans work under stressful conditions and environments, which may predispose them to some behaviors such as drinking, violence, and smoking [3]. Furthermore, construction artisans belong mostly to the lower socioeconomic status (SES) in Nigeria. Despite this evidence that tobacco use is highly concentrated in populations with low SES [11] such as construction artisans, no study has been conducted to provide insight into the smoking behavior of this population in Nigeria, the most populous country in Africa [5]. Therefore, the aim of this study was to assess the prevalence of smoking and associated factors among construction artisans in a state in Nigeria.

## Methods

**Study design and setting:** this is a cross-sectional study carried out in Ekiti State, one of the 36 States of Nigeria. It is in the southwestern part of the country. The State has 16 Local Government Areas (LGAs) and three senatorial districts. Ekiti State is a culturally homogenous State, inhabited predominantly by the Ekiti sub-ethnic group of the Yorubas. Ekiti State is still developing, and many construction sites are ongoing, in various stages of development in different parts of the State.

**Study population:** artisanship is very common, in carpentry, bricklaying, and welding, in Ekiti State making the state a good site for this study. The target population was artisans, journeymen, and their apprentices who were 18 years and above, working and living in the state. Artisans who did not give consent were excluded from the study. A multi-stage sampling technique was used. In the first stage, two LGAs were selected by simple random sampling from each of the three senatorial districts of the State, giving six LGAs. Thereafter, from the list of registered artisans for carpenters and bricklayers in these six LGAs, a simple random sampling was done to obtain participants. The minimum sample size was determined using Fisher´s formula [12].


$$n=\frac{\left(Z^{2}P\left(1-P\right)\right)}{E^{2}}$$

Where n= minimum sample size, z= standard Normal Deviate corresponding to the confidence level of 95% for a tailed test= 1.96, P=proportion of tobacco use amongst informal sector workers in a previous study= 9.6% [3], E= level of error one is prepared to accept when estimating the proportion in the sample= 5%. n=133.35. To compensate for non-response, ns= n 0.90, n= calculated sample size and ns= sample size to compensate for non-response. ns=148.1. So, the sample size after adjusting for non-response n= 149.

**Data collection:** a survey of pre-tested, semi-structured questionnaires was administered to construction artisans after completing an informed consent to participate in the study. The questionnaire was adapted from the contents of validated WHO and Centre for Disease Control and Prevention´s (CDC) Global Adult Tobacco Survey (GATS) and the WHO MONICA project protocol for recording smoking history [9]. The pre-test of the questionnaire was done on 10% of the participants in Ido/Osi Local Government Area, which was not included in the study.

### Definitions

**Dependent variable:** lifetime tobacco use. The lifetime tobacco use was assessed with an adapted question from GATS: “have you ever used tobacco” and the responses were “Yes” or “No”. For the analysis, while “Yes” was coded as “1”, “No” was coded as “0” (zero).

**Independent variables:** these variables were selected because prior studies suggest they are predictors of tobacco use. They include age level of education, marital status, religion, tribe, and years of experience as an artisan. Age was assessed as “age as at last birthday” and the responses were “less than or 20 years, 21-30 years, 31-40 years, > 40 years” This was coded as “0,1,2,3” respectively. The level of education was determined with the question “What is your highest level of education?” and the responses were “no formal education, primary education, secondary education, and tertiary education”. This was coded as “0,1,2,3, and 4” respectively”. Religion was ascertained with the question “What is your religion?” and the responses were “Christianity, Islam, Traditional, and others (specify)”. This was coded as “0,1,2,3” respectively. Finally, years of experience as an artisan was ascertained with the question “How long have you been doing this job?” and the responses were “less than a year, 1-5 years, 6-10 years, 11-20 years, and more than 20 years” and these were coded as “0,1,2,3, and 4” respectively. All these variables were recoded as mutually excluded categories for the analysis.

**Statistical analysis:** data collection and editing were conducted manually to detect omissions and to ensure uniform coding. The data was entered into a computer and analysis was completed using Statistical Package for the Social Sciences (SPSS) version 21. All categorical variables, frequency tables, and cross-tabulations were generated. A chi-square test was used to determine the statistical significance and association between dependent and independent variables. A multivariable analysis using logistic regression was carried out to determine the factors associated with smoking. A logistic regression analysis was preferred because lifetime tobacco use is a dichotomous variable (Yes or No) and many independent variables can be tested for association in this analysis. The level of significance was determined at a p-value < 0.05 with a 95% confidence level (CI). Univariable analysis was conducted to determine the demographic distribution of the artisan population. Thereafter, a bivariable analysis was done to determine the differences and statistically significant associations between dependent variables- lifetime tobacco use (Y/N) and independent variables- including age, sex, race, region, and years of experience, and the p-values were reported. Variables with p < 0.05 were included in the full logistic regression model for the multivariate analysis then backward elimination was used to select the final model predicting lifetime tobacco use among artisans.

**Ethical considerations:** the pretested questionnaires with participants' information sheets and informed consent forms were administered directly to the artisans at their place of work by the researcher. Participants who agreed to participate by signing the informed consent form were recruited. Research approval was obtained from the Ethics and Research Review Committee of the Federal Teaching Hospital, Ido Ekiti, Ekiti State, Nigeria. The reference number was ERC/2018/08/07/132A.

## Results

A total of 228 construction artisans responded well to the survey question. Most of the respondents were between the ages of 21-30 years. All respondents were males. Almost three-fifths (57%) of the respondents were married while the remaining 43% were either single (33.3%), divorced separated (6.6%), or widowed (3.1%). Two-fifths (39.9%) of the respondents were apprentices but a third (33.8%) of the respondents had more than 20 years of work experience. Among the respondents, 19.3% had ever smoked a cigarette in the last 12 months while more than 80% of the construction artisans (CA) had not. More than half (53.2%) lifetime smokers started smoking before the age of 20 years and two-thirds (65.9%) of the construction artisans admitted to having started the habit because of friends. Half (50%) of the ever-smokers still indulge in the habit, and 90% of them continued in this habit for the pleasure they derived from smoking (Table 1). Furthermore, 86.4% admitted to the fact that the smoking habit of their colleagues enticed them to smoke cigarettes. All the respondents were aware that smoking is injurious to health, and all had to access health information on tobacco use. However, 90.9% of the construction artisans who currently smoke were willing to quit but 9.1% did not want to quit smoking because feel they were addicted and could not imagine their life without a cigarette (Table 1).

**Table 1:** the smoking history of construction artisans in this study

Variable	Frequency N = 44	Percentage (%)
**Ever smoked a cigarette in the last 12 months**		
Yes	44	19.3
No	184	80.7
**Total= 228**		
**Age at first smoke**		
< 20 years	23	52.3
≥ 20 years	21	47.7
**Reasons you started**		
Friends	29	65.9
Adventure	15	34.1
**Currently, smoke**		
Yes	22	50.0
No	22	50.0
**Reason for still indulging (n=22)**		
Pleasure	20	90.9
Recognition	2	9.1
**Average no of sticks per day (n=22)**		
1 – 5	15	68.2
> 5	7	31.8
**Nature of work demands to smoke**		
Yes	14	31.8
No	30	68.2
**No of my co-workers smoking**		
< 5	36	81.8
≥ 5	8	18.2
**Aware smoking is injurious to your health**		
Yes	42	95.5
No	2	4.5
**A co-worker's smoking habit entices you to smoke**		
Yes	38	86.4
No	6	13.6
**Want to quit smoking (n=22)**		
Yes	20	90.9
No	2	9.1

**Table 2:** socio-demographic factors associated with ever smokers among respondent construction artisans

Variable	Ever use tobacco				
	**Yes n (%)**	**No n (%)**	**χ2**	**Df**	**P-value**
**Age group (in years)**					
≤ 20	13 (17.1)	63 (82.9)	18.9	3	**1** <0.001
21 – 30	7 (10.8)	58 (89.2)			
31 – 40	19 (41.3)	27 (58.7)			
> 40	5 (12.2)	36 (87.8)			
**Currently in school**					
Yes	26 (25.2)	77 (74.8)	4.263	1	0.039
No	18 (14.4)	107 (85.6)			
**Highest educational level**					
None	1 (8.3)	11 (91.7)	8.537	4	0.074
Primary	1 (3.1)	31 (96.9)			
Secondary	23 (21.5)	84 (78.5)			
Vocational	6 (20.7)	23 (79.3)			
Tertiary	13 (27.1)	35 (72.9)			
**Marital status**					
Single	13 (17.1)	63 (82.9)	8.306	3	0.04
Married	22 (16.9)	108 (83.1)			
Divorced/Separated	7 (46.7)	8 (53.3)			
Widowed	2 (28.6)	5 (71.4)			
**Marriage type (if ever married)**					
Monogamous	19 (16.1)	99 (83.9)	5.988	1	0.014
Polygamous	12 (35.3)	22 (64.7)			
**Religion**					
Christianity	33 (18.2)	148 (81.8)	0.978	2	0.613
Islam	8 (25.8)	23 (74.2)			
Traditional	3 (18.8)	13 (81.2)			
**Tribe**					
Yoruba	35 (18.2)	157 (81.8)	1.126	3	0.771
Hausa	2 (20.0)	8 (80.0)			
Igbo	6 (27.3)	16 (727)			
Others	1 (25.0)	3 (75.0)			
**Apprentice**					
Yes	7 (7.7)	84 (92.3)	13.1	1	<0.001
No	37 (27.0)	100 (73.0)			
**Years of experience**					
<1	4 (6.7)	56 (93.3)	9.823	4	<0.00
1-5	6 (17.6)	28 (82.4)			
6-10	8 (22.2)	28 (77.8)			
11-20	5 (23.8)	16 (76.2)			
>20	21 (27.3)	56 (72.7)			

**Figure 1:**
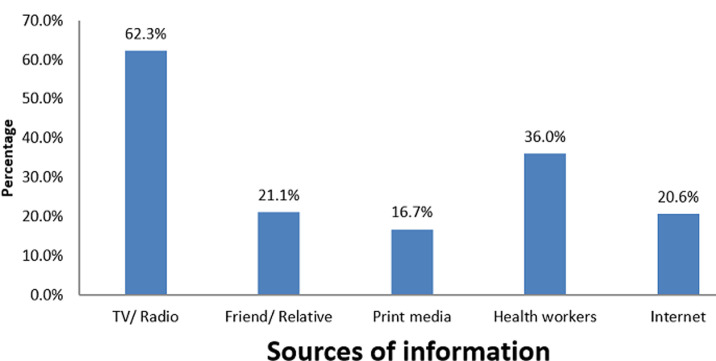
respondent's sources of tobacco-related health information

**Factors associated with tobacco use:** there was a statistically significant association between age and tobacco use amongst construction artisans (p<0.001) Table 2). Construction artisans within the age range 31-40 and those < 20 years smoke more than those in other age groups. There was also a significant association between ever smoking and not being an apprentice (p<0.001). Construction artisans who had more years of experience in their occupation smoked more than those who were apprentice. In a similar vein, there was a statistically significant association between years of experience and tobacco use (p=0.044). Construction artisans with over 20 years of experience smoke more than those with fewer years of experience. Construction artisans who are within the age range of 31-40 years are 3.4 times more likely to use tobacco than those < 20 years (aOR: 3.410, 95% CI: 1.476-7.878; p= 0.003) (Table 3). Similarly, those who are currently in school and those who are divorced/separated are twice as likely (aOR: 2.007, 95% CI: 1.026-3.927; p= 0.039) and 4.2 times more likely (aOR: 4.240, 95% CI: 1.307-13.759; p= 0.012) to use tobacco than those not in school and those single, respectively. Furthermore, construction artisans who were not apprentices were 4.4 times more likely to smoke than those who were apprentices (aOR: 4.440, 95% CI 1.882-10.475; p < 0.001). More than three-fifths (62.3%) of the respondents get health information on tobacco use from TV/radio, and print media was found to be the least explored means of getting health information. Also, among the respondents, 36% get health information from health workers while 21% and 20.6% get health information from friends/relatives and the Internet, respectively (Figure 1). Furthermore, when asked about smoking restrictions at the worksite, almost two-thirds of respondents (64.9%) said there was no smoking restriction at work sites.

**Table 3:** adjusted odd ratios for the associated factors of ever smokers among respondents

Variable	AOR	95% CI for AOR		
		Lower	Upper	P-value
**Age group (in years)**				
≤ 20	1.000			
21 – 30	0.585	0.218	1.567	0.282
31 – 40	3.410	1.476	7.878	0.003
> 40	0.673	0.222	2.042	0.482
**Currently in school**				
Yes	2.007	1.029	3.927	0.039
No	1.000			
**Marital status**				
Single	1.000			
Married	0.987	0.465	2.096	0.973
Divorced/Separated	4.240	1.307	13.759	0.012
Widowed	1.939	0.339	11.101	0.451
**Marriage type (if ever married)**				
Monogamous	1.000			
Polygamous	2.842	1.205	6.701	0.040
**Apprentice**				
Yes	1.000			
No	4.440	1.882	10.475	<0.001
**Years of experience**				
< 1	1.000			
1 – 5	3.000	0.782	11.504	0.097
6 – 10	4.000	1.109	14.432	0.026
11 – 20	4.375	1.050	18.234	0.031
> 20	5.250	1.693	16.278	0.002

## Discussion

This study aimed to assess the prevalence of smoking and associated factors among construction artisans in Ekiti State in Nigeria. The estimated prevalence of tobacco use among adults aged 15 years and older was 22.3% in 2020 year, which translated to 1.7 billion [5,13]. Thus, due to its large population size, Nigeria has the largest crude number of smokers among all the countries in sub-Saharan Africa. Nonetheless, smoking in Nigeria continues to rise and the WHO has projected that adult smoking in the country will increase by 5% by 2025. Further, studies have shown disparities in the prevalence of tobacco use and tobacco-induced diseases exist, with tobacco use increasingly being concentrated in populations with low SES [5,14] such as blue-collar workers [10]. However, these disparities have not been explored in Nigeria. This study found that 19.3% were lifetime smokers, which is higher than the prevalence of ever smokers in the general population (3.9%) [15]. Similarly in Morocco, the prevalence of smoking among blue-collar workers was 28.5% [7], which was also higher than the prevalence in the population, which was 18% [16]. Moreover, a survey analysis of national data between 1992 and 2007 in the United States also gave the prevalence of smokers among construction artisans in the United States to be 38% [9] and in Brazil, a cross-sectional survey among 418 male construction workers ever smoking prevalence was found to be 72.4% [17]. In these countries, the prevalence of ever-used tobacco in the general population was 30% and 56.9% [9,17] in the United States and Brazil respectively. Therefore, the prevalence of ever-used tobacco was particularly higher amongst this group of workers.

However, the prevalence of ever-smoke amongst construction artisans in this study which was 19.3% is quite lower when compared to the prevalence of ever-smoke amongst CA in Brazil which was 72.4% [17]. This may not be unconnected to the fact that the prevalence of smoking in the general population in Brazil (56.9%) [17] was far higher than the prevalence of ever smokers in the general population in Nigeria (3.9%) [5]. It must also be stressed that the prevalence of smokers in the general population in Nigeria was low possibly because some adults for cultural, spiritual, and social reasons would not admit having ever used tobacco. In this study, several factors were identified to be associated with smoking. The age of artisans, marital status, and marriage type of CA, whether construction artisans were presently in school, years of experience, and level of expertise of the CA were found to be significantly associated with tobacco use amongst construction artisans. Artisans who are within the age range of 31-40 years were 4.4 times more likely to smoke than those < 20 years. This was also in line with a study done in Morocco by Nejjari *et al*. [16] and Jagoe *et al*. in Tanzania [18] They found that men who were between 30-39 years of age have a high risk of tobacco use when compared with other population age groups. Barbeau *et al*. [19] and Okechukwu *et al*. [20] in their respective studies, also highlighted a significant association between increasing age and smoking behavior among construction artisans [19,20]. Furthermore, construction artisans who were divorced/separated were 4.2 times more likely to use tobacco. Similarly, a population survey in Tanzania shows that divorced men were more likely to be smokers than married men. Liu *et al*. (2015) in a cross-sectional study amongst 5,380 migrant workers in China not only found 8.8 times increased risk of smoking in those who work in the construction sector but also found 2.2 times increased risk of smoking among these workers who were divorced [21].

This could mean that the physical stress of the construction work (which 31.8% of smokers admitted to) and the smoking behavior of colleagues (which 86.4% of smokers admitted to) increases their uptake of the smoking habit on the one hand, and emotional and marital stress can increase their propensity to smoke on the other hand [21]. Moreover, artisans with more than five years of work experience were more likely to smoke when compared with those with fewer years of working experience. In this study, logistic regression analysis showed that construction artisans with 6-10 years, 11-20 years, and > 20 years of working experience were four, five, and six times, respectively more likely to have ever smoked than those with less than one of year experience. This was also in tandem with the findings of Gavioli *et al*. [17] in a cross-sectional study of substance use amongst construction artisans in Brazil [17]. They opined that construction artisans with more than 10 years of working experience had an increased risk of ever smoking tobacco. It can then be concluded that the longer an artisan stays on the job, the higher his chances of taking up a smoking habit. This could be because he would be more exposed to the stress and strain of the job which may demand the use of a substance such as tobacco to relieve. Moreover, the more years an artisan spends on his job, the larger his circle of friends who are his co-workers and who might also be a smoker. With time, he might also take up the smoking habit just to conform to his co-workers.

It is also striking to note that almost two-thirds (64.9%) of the respondents said there was no smoking restriction at the worksite. Asfar *et al*. [22] also noted a lack of smoking intervention that is adapted to the work environment of Hispanics/Latinos working in the construction industries in the United States [22]. Unfortunately, this lack of smoking restrictions at worksites may serve as an impetus to continue smoking. The use of tobacco cessation interventions at the worksite has been shown to impact significantly the smoking habits of construction artisans [6,22,23]. A mixed-method intervention (focus group and survey data) used amongst members of the Carpenters' District Council in the United States showed that 65% of its 144 current smoking members were willing to quit smoking post-intervention [6]. It was also noted that the majority responded positively to tailored messages and images on tobacco cessation. This study provided one of the first evidence of the exploration of the prevalence of tobacco use among artisans in Nigeria. Moreover, the study used an interview-administered questionnaire to collect primary data from the artisans, which reduced the possibility of non-response bias. However, the information on tobacco use was self-reported and this might lead to reporting bias, but self-reported estimates of tobacco use from other studies have indicated that this kind of information is still valid [23]. More so, triangulating this study with qualitative data on the perception of construction artisans on smoking would have given additional information on the smoking behavior of construction artisans.

## Conclusion

This study assessed the prevalence of tobacco use amongst construction artisans in the communities in Ekiti State. It was found that the prevalence of cigarette smoking was higher amongst construction artisans than in the general population. Furthermore, the predisposing factors for the increased prevalence of this risky behavior amongst this group of workers were also assessed; age, marital status, and years of experience were strongly associated with indulgence in tobacco usage by construction artisans. The impacts of indulging in this behavior span beyond the construction artisans themselves. There are medical, social, economic, and public health implications of this behavior to their immediate families, communities, and society at large. Therefore, individuals, communities, and governments at all levels must strive to mitigate any social and health inequalities linked with this unhealthy behavior for a better and healthier society.

### What is known about this topic

- There is higher tobacco use among blue-collar workers in other countries and some studies have associated this with the job;
- There is a higher prevalence of smokers among people from lower socioeconomic status;
- Being out of school is associated with ever-tobacco use.

### What this study adds

- The study provided a prevalence of lifetime tobacco use among artisans in Nigeria, which, to our knowledge, no research has ever been done to determine that in the country;
- Though the study shows a higher prevalence of ever-tobacco use among blue-collar workers in Nigeria compared with general popular, however, the prevalence was still lower in Nigeria when compared to other countries; this study adds sub-Saharan African evidence to the relationship between the ever-tobacco user and construction artisanship;
- Among the factors associated with ever-tobacco use, respondents who are in school are more likely to smoke; this may suggest that among blue-collar workers in sub-Saharan Africa, being in school may not necessarily be protective against ever-tobacco use.

## References

[1] Ogunsemi DR, Jagboro GO (2006). Time-cost model for building projects in Nigeria. Constr Manag Econ.

[2] Odediran SJ, Babalola O (2013). Employment structure of informal construction workers/artisans in Nigeria. Global Journal of Management and Business Research.

[3] Wahab A (2010). Stress Management among Artisans in Construction Industry in Nigeria. GJRE.

[4] Ekpo K, Okon A (2014). Vocational education and economic development in Nigeria. AFRREV IJAH Int J Arts Humanit.

[5] World Health Organization WHO global report on trends in prevalence of tobacco use 2000-2025, fourth edition.

[6] Okechukwu CA, Nguyen K, Hickman NJ (2010). Partner smoking characteristics: associations with smoking and quitting among blue-collar apprentices. Am J Ind Med.

[7] Tachfouti N, Berraho M, Elfakir S, Tachfouti N, Berraho M, Elfakir S (2010). Socioeconomic status and tobacco expenditures among Moroccans: results of the “Maroc Tabagisme” survey. Am J Health Promot.

[8] Pednekar M, Nagler E, Pawar P, Sorensen G, Narake S, Stoddard MA (2015). The prevalence of tobacco use among manufacturing workers: findings from the baseline survey of the Mumbai worksite Tobacco control study. J Prev Med.

[9] Ham DC, Przybeck T, Strickland JR, Luke DA, Bierut LJ, Evanoff BA (2011). Occupation and workplace policies predict smoking behaviors: analysis of national data from the current population survey. J Occup Environ Med.

[10] Sreeramareddy CT, Pradhan PM, Sin S (2014). Prevalence, distribution, and social determinants of tobacco use in 30 sub-Saharan African countries. BMC Med.

[11] Hiscock R, Bauld L, Amos A, Fidler JA, Munafò M (2012). Socioeconomic status and smoking: a review. Ann N Y Acad Sci.

[12] Bruce N, Pope D, Stanistreet D (2018). Quantitative methods for health research: a practical interactive guide to epidemiology and statistics. John Wiley and Sons.

[13] (2020). Worldometer World Demographics.

[14] John RM, Mamudu HM, Liber AC (2012). Socioeconomic Implications of Tobacco Use in Ghana. Nicotine Tob Res.

[15] World Health Organization (2013). WHO report on the global tobacco epidemic, 2013: enforcing bans on tobacco advertising, promotion and sponsorship. WHO.

[16] Nejjari C, Benjelloun MC, Berraho M, El Rhazi K, Tachfouti N, Elfakir S (2009). Prevalence and demographic factors of smoking in Morocco. Int J Public Health.

[17] Gavioli A, Mathias TA de F, Rossi RM, Oliveira MLF de (2014). Risks related to drug use among male construction workers. Acta Paul Enferm.

[18] Jagoe K (2002). Tobacco smoking in Tanzania, East Africa: population-based smoking prevalence using expired alveolar carbon monoxide as a validation tool. Tob Control.

[19] Barbeau EM, Krieger N, Soobader MJ (2004). Working class matters: socioeconomic disadvantage, race/ethnicity, gender, and smoking in NHIS 2000. Am J Public Health.

[20] Okechukwu C, Bacic J, Cheng KW, Catalano R (2012). Smoking among construction workers: the nonlinear influence of the economy, cigarette prices, and antismoking sentiment. Soc Sci Med.

[21] Liu Y, Song H, Wang T, Wang T, Yang H, Gong J (2015). Determinants of tobacco smoking among rural-to-urban migrant workers: a cross-sectional survey in Shanghai. BMC Public Health.

[22] Asfar T, Caban-Martinez AJ, McClure LA, Ruano-Herreria EC, Sierra D, Gilford Clark G (2018). A cluster randomized pilot trial of a tailored worksite smoking cessation intervention targeting Hispanic/Latino construction workers: Intervention development and research design. Contemp Clin Trials.

[23] Syamlal G, King BA, Mazurek JM (2018). Tobacco product use among workers in the construction industry, United States, 2014-2016. Am J Ind Med.

